# Enzymatic Cleavage of Stx2a in the Gut and Identification of Pancreatic Elastase and Trypsin as Possible Main Cleavers

**DOI:** 10.3390/microorganisms11102487

**Published:** 2023-10-04

**Authors:** Sára Kellnerová, Silke Huber, Mariam Massri, Verena Fleischer, Klemens Losso, Bettina Sarg, Leopold Kremser, Heribert Talasz, Xiaohua He, Elisa Varrone, Maurizio Brigotti, Gianluigi Ardissino, Dorothea Orth-Höller, Reinhard Würzner

**Affiliations:** 1 Institute of Hygiene and Medical Microbiology, Medical University of Innsbruck, 6020 Innsbruck, Austria; sara.kellnerova@i-med.ac.at (S.K.); silke.huber@i-med.ac.at (S.H.); mariam.massri@i-med.ac.at (M.M.); verena.fleischer@i-med.ac.at (V.F.); 2 Institute of Analytical Chemistry and Radiochemistry, University of Innsbruck, 6020 Innsbruck, Austria; klemens.losso@mci.edu; 3 Department of Food Technology and Nutrition, MCI|The Entrepreneurial School, 6020 Innsbruck, Austria; 4 Protein Core Facility, Institute of Medical Biochemistry, Center of Chemistry and Biomedicine, Medical University of Innsbruck, 6020 Innsbruck, Austria; bettina.sarg@i-med.ac.at (B.S.); leopold.kremser@i-med.ac.at (L.K.); heribert.talasz@i-med.ac.at (H.T.); 5 Western Regional Research Center, U.S. Department of Agriculture, Agricultural Research Service, Albany, CA 74710, USA; xiaohua.he@ars.usda.gov; 6 Department of Medical and Surgical Sciences, School of Medicine, University of Bologna, 40126 Bologna, Italy; elisa.varrone2@unibo.it (E.V.); maurizio.brigotti@unibo.it (M.B.); 7 Center for HUS Prevention, Control and Management at Pediatric Nephrology, Dialysis and Transplant Unit, Fondazione IRCCS Ca’ Granda, Ospedale Maggiore Policlinico, 20122 Milan, Italy; ardissino@centroseu.org; 8 MB-LAB–Clinical Microbiology Laboratory, 6020 Innsbruck, Austria

**Keywords:** enterohemorrhagic *Escherichia coli* (EHEC), EHEC-associated hemolytic uremic syndrome (eHUS), Shiga toxin 2a (Stx2a), trypsin, furin, chymotrypsin-like elastase 3B (CELA3B)

## Abstract

Shiga toxins (Stxs), especially the Stx2a subtype, are the major virulence factors involved in enterohemorrhagic *Escherichia coli* (EHEC)-associated hemolytic uremic syndrome (eHUS), a life-threatening disease causing acute kidney injury, especially in children. After oral transmission and colonization in the gut, EHEC release Stx. Intracellular cleavage of the Stx A subunit, when followed by reduction, boosts the enzymatic activity that causes damage to targeted cells. This cleavage was assumed to be mostly mediated by furin during Stx intracellular trafficking. To investigate whether this cleavage could occur in the intestine, even prior to entering target cells, Stx2a A subunit structure (intact or cleaved) was characterized after its exposure to specific host factors present in human stool. The molecular weight of Stx2a A subunit/fragments was determined by immunoblotting after electrophoretic separation under reducing conditions. In this study, it was demonstrated that Stx2a is cleaved by certain human stool components. Trypsin and chymotrypsin-like elastase 3B (CELA3B), two serine proteases, were identified as potential candidates that can trigger the extracellular cleavage of Stx2a A subunit directly after its secretion by EHEC in the gut. Whether the observed cleavage indeed translates to natural infections and plays a role in eHUS pathogenesis has yet to be determined. If so, it seems likely that a host’s protease profile could affect disease development by changing the toxin’s biological features.

## 1. Introduction

Infections with enterohemorrhagic *Escherichia coli* (EHEC) are the most prominent cause of pathogen-induced hemolytic uremic syndrome (HUS), the latter comprising microangiopathic anemia, thrombocytopenia, and acute renal failure [1]. eHUS is a life-threatening condition, especially in childhood, with a mortality rate of 3–5% [1,2]. The major virulence factors involved in EHEC-associated hemolytic uremic syndrome (eHUS) are Shiga toxins (Stxs) [3,4]. Currently, the diagnosis of such EHEC infections is determined by examining the patient’s stool for Stx or *stx* genes and culturing the causative bacteria [1,5,6,7,8].

Following oral transmission, EHEC colonize the gut and releases Stxs, which subsequently transfer to the bloodstream to reach their target organs, primarily the kidneys [3,9]. Consequently, Stxs bind to globotriaosylceramide Gb3Cer or Gb4Cer receptors, found on human kidney cells and in other organs, including intestine and brain [10,11,12,13], resulting in the internalization of Stxs and consequent inhibition of protein synthesis [14]. Therefore, Stxs are classified as ribosome-inactivating proteins and in addition are further categorized into two immunologically distinct types, Stx1 and Stx2. The latter, and in particular the Stx2a subtype, is frequently linked to severe disease progression in humans [15,16].

Stxs have an AB5 structure consisting of a single enzymatically active A subunit (~32 kDa) and five non-covalently bound B subunits (each ~7.7 kDa). While the B-pentamer is responsible for the binding to respective receptors, the A subunit is responsible for the inhibition of protein synthesis within targeted cells [17,18]. During intracellular trafficking, the Stx A subunit is cleaved into two fragments, A1 (~27.5 kDa) and A2 (~4.5 kDa). Nevertheless, the A1 and A2 fragments remain linked via a disulfide bridge until its reduction in the endoplasmic reticulum (ER). The A1 fragment can then translocate to the cytosol where it functions as a protein synthesis inhibitor. Furin, an mainly intracellular protease, has been proposed to be the main enzyme responsible for the cleavage of the A subunit [19]. Nonetheless, other proteases, including trypsin, calpain, and mouse pancreatic elastase, have been demonstrated to induce cleavage of Stxs [18,20,21]. Interestingly, mouse pancreatic elastase, highly homologous to human chymotrypsin-like elastase 3B (CELA3B), was reported to induce cleavage of the Stx2d A2 fragment, resulting in increased virulence of the toxin in mice [22,23]. However, this effect has not been observed for other Stx subtypes.

Recently, the structure of the Stx2a A subunit, intact or cleaved, was shown to determine the toxin’s interaction with human blood components. While both toxin forms (uncleaved and cleaved) are fully active in intoxicating sensitive cells through Gb3Cer, only the intact Stx2a (in particular the Stx2a A subunit) was found to bind to neutrophils via Toll-like receptor 4 (TLR4) and to allow the formation of platelet–leukocyte complexes [24]. Cleaved Stx2a, unlike the intact form, was able to bind complement regulator factor H (fH) [24,25] abrogating its protective effects for target endothelial cells, and thus exacerbating complement-mediated injuries. This cleavage, however, was artificially induced during the toxin’s purification procedure.

The main aim of the present study was to investigate whether the cleavage of Stx2a A subunit could potentially occur in the course of an EHEC infection prior reaching the target cells, with special focus on the intestine. To resemble the intestinal environment, stool samples of healthy and diseased individuals were exposed to Stx2a. The form of Stx (uncleaved or cleaved) was determined by immunoblotting and, furthermore, particular cleavage sites were investigated, leading to the identification of potential Stx cleavers present in human stool. Understanding the structure–function relationship of Stxs during EHEC infections and its role in eHUS pathogenesis may eventually aid in the development of efficient preventative or therapeutic strategies.

## 2. Materials and Methods

### 2.1. Stx2a Production and Purification

Stx2a production and purification was performed as described earlier [24] with two modifications including eliminating the sonication step and performing all purification steps at 4 °C instead of room temperature (RT) in order to obtain uncleaved Stx2a. The purity of the protein was evaluated by SDS-PAGE.

### 2.2. Sampling and Fractioning of Stool

Stool from healthy human volunteers (*n* = 12, 0–99 years) was kindly donated and collected in accordance with the guideline of the Declaration of Helsinki after written consent was given by donors or their legal guardians. After collection, stool samples were stored at 4 °C until further processing. Stool samples were diluted 25% (*w*/*v*) in 0.9% NaCl and thoroughly mixed. Subsequently, stool suspension was centrifuged (687× *g*, 5 min, RT) and the supernatant was sterile-filtered (0.2 µm; Sartorius, Göttingen, Germany), and stored at −80 °C until further analysis.

Size exclusion chromatography was used to fractionate sterile-filtered supernatant from stool of healthy individuals. Fractionation was performed with an ACQUITY Arc system (Waters Corp, Milford, MA, USA) equipped with a 2998 PDA detector and a WFM-A fraction collector. Separation was performed within 10 min on a XBridge Protein BEH SEC 200 Å column (2.5 µm, 4.6 × 300 mm; Waters Corp, Milford, MA, USA) at 35 °C with isocratic solvent composition of 150 mM NaCl and 25 mM Na_3_PO_4_ at pH 7.4. The flow rate for the separation was set to 0.7 mL/min with an injection volume of 5 µL. Detection was performed at 280 nm. Each major peak was collected in a separate vial for further analysis. Fractions were recovered in the buffer described above and kept at −80 °C until further analysis.

Residual stool samples from pediatric patients admitted to the Center for HUS Prevention, Control and Management at Pediatric Nephrology, Dialysis and Transplant Unit, Fondazione IRCCS Ca’ Granda, Ospedale Maggiore Policlinico, Milan, Italy, with a confirmed EHEC infection suffering from different disease progressions including bloody diarrhea and eHUS (*n* = 12), were collected. The sample collection was performed in respect to the guideline of the Declaration of Helsinki and after written consent was given by the patient’s legal guardian. Stool samples were sent to our institution. Solid samples (*n* = 2) and one liquid sample with too little volume were excluded to maintain comparability between patient samples. Remaining liquid stool samples (*n* = 9) were diluted 1:10 in 0.9% NaCl. Subsequently, patient samples were treated and analyzed equally as samples derived from healthy individuals.

### 2.3. Cleavage of Stx2a A Subunit by Trypsin or Furin

Artificial cleavage of Stx2a A subunit was induced by incubation of Stx2a with trypsin or furin (both Sigma-Aldrich, St. Louis, MO, USA) as described previously [24]. Artificially cleaved Stxs were used to localize the cleavage site, after SDS-PAGE separation, by mass spectrometry analysis of amino acid sequence, as described in Section 2.7. Trypsin-cleaved (T-cl.) Stx2a served as a positive control for immunoblotting.

### 2.4. Incubation of Stx2a with Human Stool Supernatant or Its Fractions with or without Protease Inhibitors

Stored sterile-filtered supernatants of human stool were diluted 1:20 (*v*/*v*) in 0.9% NaCl. In absence of any protease inhibitors, diluted supernatants or their fractions (10 µL) were supplemented with Stx2a (0.3 µg in 1% (*w*/*v*) bovine serum albumin (BSA)/phosphate buffered saline (PBS)) and incubated (10, 15 or 30 min as indicated, 37 °C) with continuous shaking. Stx2a or T-cl. Stx2a (both 0.3–0.8 µg) in 0.9% NaCl (10 µL) were treated equally and used as controls. In the setup including protease inhibitors, prior to the addition of Stx2a, 1 µL of 10, 20 or 200 mM 4-(2-Aminoethyl)-benzolsulfonylfluorid (AEBSF) (Sigma-Aldrich, St. Louis, MO, USA), 0.5 mM Bestatin (Sigma-Aldrich), 0.16 mM E-64 (Sigma-Aldrich), 0.1 mM Pepstatin A (Sigma-Aldrich), 0.2 mM Leupeptin (Merck, Darmstadt, Germany), 0.0008 mM Aprotinin (Merck) or protease inhibitor cocktail (Sigma-Aldrich) was added to diluted stool supernatants (10 µL) and incubated (10–15 min, 37 °C, shaking). After this pre-incubation, Stx2a was added and samples were treated under the same conditions as the setup without protease inhibitors. Samples were separated by SDS-PAGE and the molecular size of the Stx2a A subunit/fragments was successively assessed by Western blot as described in Section 2.6.

In order to assess the exact cleavage site of Stx2a A subunit after contact with human stool components, sterile-filtered human stool supernatant were diluted 1:2 or 1:4 (*v*/*v*) in 0.9% NaCl (20 µL) and incubated (2 h, 37 °C, shaking) with Stx2a (5 µg). After SDS-PAGE separation, the localization of the cleavage site was analyzed by mass spectrometric analysis of the amino acid sequence, as explained in Section 2.7.

### 2.5. Incubation of Stx2a with Recombinant CELA3B

To investigate the Stx2a A subunit/fragments size under the influence of CELA3B, 0.125, 1, or 2 µg of recombinant human CELA3B (Cloud-Clone, Houston, TX, USA) diluted in buffer (20 mM Tris-HCl 150 mM NaCl pH 8) was incubated with Stx2a (0.3 µg) in 1% BSA in PBS (*w*/*v*) (2 or 16 h, 37 °C, shaking). Stx2a diluted only in buffer incubated under the same conditions served as the control. Samples were separated in an SDS-PAGE and the molecular size of the Stx2a A subunit was subsequently assessed by Western blotting, as described in Section 2.6.

In order to assess the exact cleavage site of Stx2a A subunit after contact with recombinant human CELA3B (Cloud-Clone), Stx2a (5 g) was incubated with CELA3B (2 µg) (2 h, 37 °C). After SDS-PAGE separation, the mapping of the amino acids, where cleavage took place, was analyzed by mass spectrometry, as explained in Section 2.7.

### 2.6. Determination of the Structure of Stx2a A Subunit by Immunoblotting

Stx2a exposed to CELA3B, human stool supernatants or fractions of the stool samples with a loading volume of 10 µL per pocket was separated by electrophoresis (SDS-PAGE) using in-house 16% polyacrylamide gels under reducing conditions. Next, the transfer of proteins to polyvinylidene fluoride (PVDF) membranes (Bio-Rad, Hercules, CA, USA) was performed using the Trans-Blot^®^ Turbo™ Transfer System (Bio-Rad). Membranes were blocked (5% BSA in TBST (*w*/*v*), 1 h, RT) and incubated with non-commercialized mouse polyclonal antibodies against Stx2a (1:7.5 (*v*/*v*) with 0.5% BSA in TBST (*w*/*v*), gifted by Prof. Dr. Peter Garred, Copenhagen, Denmark (overnight, 4 °C, rolling). After washing, horseradish peroxidase (HRP)-conjugated rabbit anti-mouse IgG antibody (Dako, Santa Clara, CA, USA), diluted 1:2500 (*v*/*v*) in 5% BSA TBST (*w*/*v*), was applied to the membranes (1 h, RT, shaking). Lastly, using ImageQuant LAS 4000 (GE Healthcare, Munich, Germany), the bands corresponding to the A subunit of Stx2a were detected by enhanced chemiluminescence (ECL) after applying the respective substrates (Bio-Rad). Stx2a A subunit cleavage (presence or absence) was interpreted based on the protein’s migration compared to the prestained protein ladder (Bio-Rad) as well as to the reference proteins, Stx2a and T-cl. Stx2a.

### 2.7. Identification of the Cleavage Position of the A Subunit of Stx2a by Components Present in Human Stool

Stx2a exposed to trypsin, furin, CELA3B, or distinct human stool supernatants was separated by electrophoresis (SDS-PAGE) using home-made 16% polyacrylamide gels under reducing conditions. After electrophoresis, gels were incubated with Coomassie brilliant blue staining solution (25 mL, 1 h, RT) followed by Coomassie-distaining solution (25 mL, overnight, RT, solution exchanged after first 1–2 h). Coomassie-stained gel bands harboring Stx2a A subunit or A1 fragment were excised from SDS-PAGE gels, reduced with dithiothreitol, alkylated with iodoacetamide and digested with trypsin (Promega, Waldorf, Germany) or chymotrypsin (Sigma-Aldrich) as previously described [26]. Digested samples were analyzed using an UltiMate 3000 RSLCnano-HPLC system coupled to a Q Exactive HF or an Orbitrap Eclipse mass spectrometer (both Thermo Scientific, Bremen, Germany) via a Nanospray Flex ionization source. The peptides were separated on a homemade fritless fused-silica micro-capillary column (100 µm i.d. × 280 µm o.d. × 16 cm length) packed with 2.4 µm reversed-phase C18 material. Solvents for HPLC were 0.1% formic acid (solvent A) and 0.1% formic acid in 85% acetonitrile (solvent B). The gradient profile was as follows: 0–4 min, 4% B; 4–57 min, 4–35% B; 57–62 min, 35–100% B; and 62–67 min, 100% B. The flow rate was 300 nl/min.

The Q Exactive HF mass spectrometer was operated as described before [27].

The Orbitrap Eclipse mass spectrometer was operated in the data-dependent mode with a cycle time of one second. Survey full scan MS spectra were acquired from 375 to 1500 mass-to-charge ratio (*m*/*z*) at a resolution of 240,000 with an isolation window of 1.2 *m*/*z*, a maximum injection time (IT) of 50 ms, and automatic gain control (AGC) target 400,000. The MS2 spectra were measured in the Orbitrap analyzer at a resolution of 15,000 with a maximum IT of 22 ms, and AGC target or 50,000. The selected isotope patterns were fragmented by higher-energy collisional dissociation with normalized collision energy of 28.

Data analysis was performed using Proteome Discoverer 2.5 (Thermo Scientific) with the search engine Sequest. Enzyme specificity was set to semi-specific or unspecific, and the raw files were searched against Uniprot human database (20,528 entries), and a database containing Shiga toxin and the most common protein contaminants like keratins, BSA, trypsin, etc. (536 sequences). Precursor and fragment mass tolerance was set to 10 ppm and 0.02 Da, respectively, and up to two missed cleavages were allowed. Carbamidomethylation of cysteine and oxidation of methionine were set as variable modification.

### 2.8. Detection of Chymotrypsin-like Elastase 3B (CELA3B) by Immunoblotting

The stored sterile-filtered supernatant of human stool was diluted 1:5 in 0.9% NaCl. CELA3B in human stool supernatants was separated by electrophoresis (SDS-PAGE) using in-house 16% polyacrylamide gels under non-reducing conditions. Next, the transfer of proteins to polyvinylidene fluoride (PVDF) membranes (Bio-Rad, Hercules, CA, USA) was performed. Membranes were blocked (5% BSA in TBST (*w*/*v*), 1 h, RT) and incubated with mouse monoclonal antibodies against CELA3B (2 µg/mL; Sigma-Aldrich) in 0.5% BSA in TBST (*w*/*v*) (overnight, 4 °C, rolling). After washing, horseradish peroxidase (HRP)-conjugated rabbit anti-mouse IgG antibody (Dako, Santa Clara, CA, USA), diluted 1:2500 (*v*/*v*) in 5% BSA TBST (*w*/*v*), were applied to the membranes (1 h, RT, shaking). Bands corresponding to CELA3B were detected by enhanced chemiluminescence (ECL) after applying the respective substrate (Bio-Rad) using the ImageQuant LAS 4000 (GE Healthcare). Recombinant human CELA3B (Cloud-Clone) was used as control.

## 3. Results

### 3.1. Enzymes Present in Human Stool Cleave the A Subunit of Stx2a In Vitro

**Figure 1:**
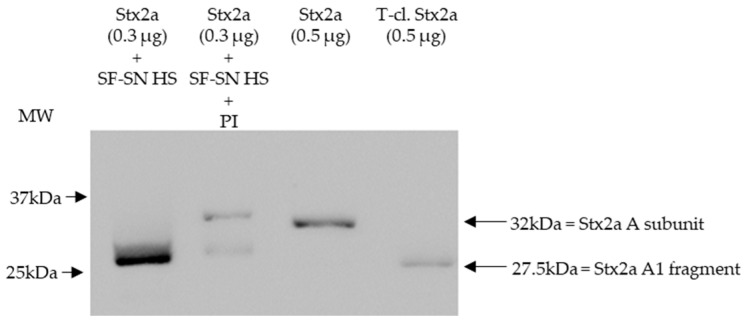
Shiga toxin 2a (Stx2a) A subunit and/or its A1 fragment after exposure to stool specimens of a healthy individual. This representative immunoblot shows the form (intact or cleaved) of Stx2a after its incubation (10 min) with sterile-filtered supernatant derived from human stool (SF-SN HS) with or without a protease inhibitor cocktail (PI). Pure Stx2a and trypsin-cleaved (T-cl.) Stx2a served as references. Bands showcasing the whole A subunit or the A1 fragment of Stx2a are indicated by arrows (the A2 fragment (~4.5 kDa) would be too short to be detected). Molecular weight (MW) is indicated in kilodaltons (kDa) based on the migration of the molecular markers.

To mimic the physiological conditions present after an EHEC infection, in which Stx2a is released by bacteria and exposed to components present in the intestine, Stx2a was incubated in vitro with supernatant derived from stool of healthy individuals and its structure (intact or cleaved) was then analyzed by immunoblotting. In addition, to investigate the influence of enzymes present in stool, cleavage of Stx2a by human stool supernatant was also evaluated in the presence of a protease inhibitor cocktail containing AEBSF, Aprotinin, Bestatin, E-64, Leupeptin, and Pepstatin A. Stx2a and T-cl. Stx2a served as references to evaluate the cleavage. After incubation with different stool specimens of healthy donors (*n* = 12), the Stx2a A subunit was always completely cleaved as indicated by the presence of a 28 kDa band corresponding to the A1 fragment observed in T-cl. Stx2a. In the presence of the protease inhibitor cocktail, this cleavage was only partially observed, indicating that it was induced by specific proteolytic enzymes (Figure 1 and Figure S1).

### 3.2. Serine Protease(s) in Human Stool Are Responsible for the Cleavage of the A Subunit of Stx2a

**Figure 2:**
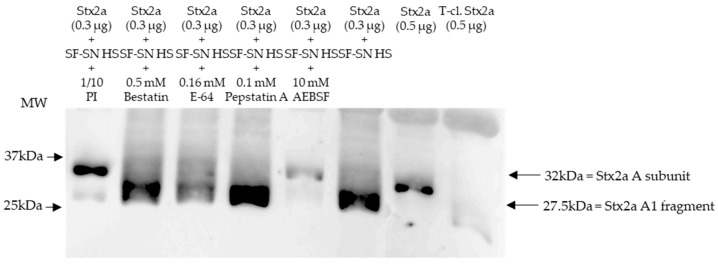
Shiga toxin (Stx) 2a A subunit and/or its A1 fragment after exposure to stool of healthy individuals in the presence of various protease inhibitors. This representative immunoblot shows the form (intact or cleaved) of Stx2a after its incubation (10 min) with sterile-filtered supernatant derived from human stool (SF-SN HS) with or without a protease inhibitor cocktail (PI). Bestatin (0.5 mM), E-64 (0.16 mM) or Pepstatin A (0.1 mM) did not prevent cleavage, while AEBSF (10 mM) did. Pure Stx2a and trypsin-cleaved (T-cl.) Stx2a served as references. Bands showcasing the whole A subunit or the A1 fragment of Stx2a are indicated by arrows. Molecular weight (MW) is indicated in kilodaltons (kDa) based on the migration of the molecular markers.

Immunoblotting of Stx2a after its incubation with human stool from healthy individuals in the presence of different protease inhibitors, which were present in the aforementioned protease inhibitor cocktail, showed that the cleavage of Stx2a A subunit by stool was inhibited when serine protease inhibitors AEBSF, Aprotinin, or Leupeptin were added (Figure 2 and Figure S2). 

Stx2a incubated with stool in the absence of protease inhibitors was used as a control, while pure Stx2a and T-cl. Stx2a were used as references to evaluate the toxin’s structure. Most importantly, inhibition of Stx2a A subunit cleavage was not observed when protease blockers other than serine protease inhibitors, although present in the aforementioned protease inhibitor cocktail–Bestatin, E-64 or Pepstatin A–, were added (Figure 2). Overall, these results suggest that a serine protease(s) is responsible for the observed cleavage of the Stx2a A subunit.

**Figure 3:**
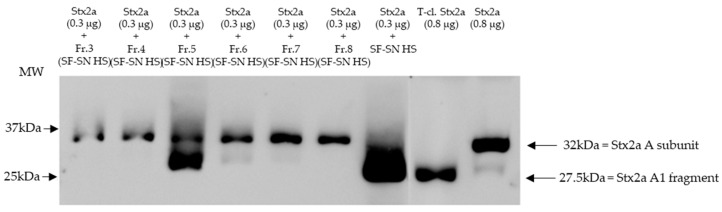
Shiga toxin (Stx) 2a A subunit and/or its A1 fragment after exposure to fractions of stool supernatant of a healthy individual. This representative immunoblot shows the form (intact or cleaved) of Stx2a after its incubation (30 min) with selected fractions (Fr. 3, 4, 6 to 8) of sterile-filtered supernatant derived from human stool (SF-SN HS) which were not able to cleave, and Fr. 5 which clearly did cleave. Pure Stx2a and trypsin-cleaved (T-cl.) Stx2a served as references. Bands showcasing the whole A subunit or the A1 fragment of Stx2a are indicated by arrows. Molecular weight (MW) is indicated in kilodaltons (kDa) based on the migration of the molecular markers.

Furthermore, sterile-filtered human stool supernatant derived from healthy donors (*n* = 6) was subjected to size exclusion chromatography producing 16 main fractions (Figure S3). The fractions were individually collected and analyzed, by immunoblotting, for their capability to induce cleavage of Stx2a A subunit. As a control, Stx2a was incubated with unfractionated human stool supernatant. Pure Stx2a and T-cl. Stx2a were used as reference for cleavage. Results provided evidence that after incubation (30 min) only one out of 16 fractions showed the ability to cleave Stx2a A subunit, namely fraction 5 (Figure 3 (with representative fractions) and Figure S4).

Overall, cleavage of Stx by incubation with fraction 5 was demonstrated for the healthy subject presented in Figure 3 and for two other healthy individuals (Figure S5a). In three other donors, fraction 5 induced at least partial cleavage when 10x-concentrated (Figure S5b,c).

To identify the enzyme responsible for Stx2a A subunit cleavage, the composition of proteins in fraction 5 was determined. Hereby, mass spectrometry analyses were performed. Overall, from the proteins detected based on abundance, unique peptides, protease specificity and the knowledge gained from experiments with protease inhibitors (Figure 3) two serine proteases, CELA3B and trypsin-2, were identified as potential candidates responsible for inducing Stx2a A subunit cleavage in the first tested healthy donor (Table S1), leading to further investigations regarding both proteins. While trypsin was present in all tested samples among the most abundant proteins (in the upper third, *n* = 4), CELA3B was detected only in three out of four. Furin was not detected.

### 3.3. Localization of the Cleavage Position of the A subunit of Stx2a Induced by Different Purified Enzymes or Components Present in Stool from Healthy Human Donors

**Figure 4:**
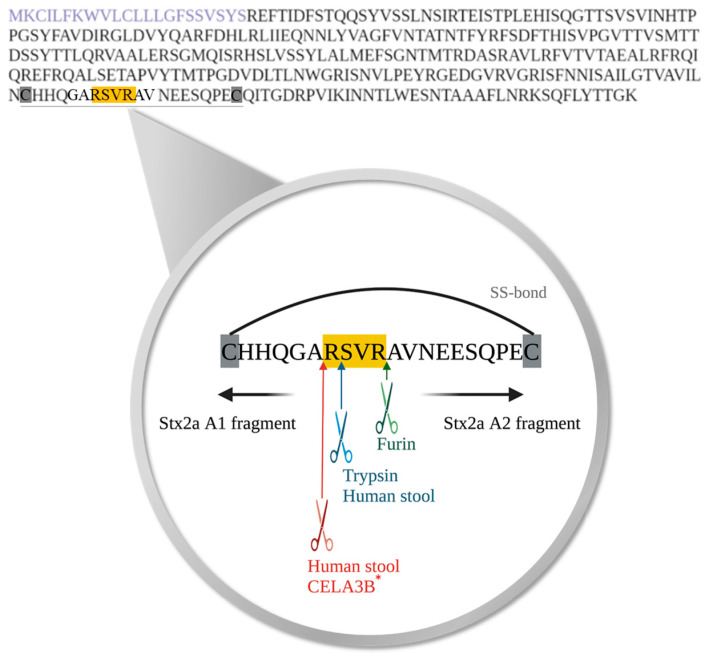
Amino acid sequence of Shiga toxin 2a (Stx2a) A subunit and the identified cleavage sites induced by specific enzymes or components present in human stool. The amino acid sequence of the A subunit of Stx2a with highlighted signal peptide (purple) is displayed. Cleavage of Shiga toxin 2a (Stx2a) A subunit results in the generation of two fragments, A1 and A2, which are held together by a disulfide bond (SS-bond) located between the two cysteine residues (highlighted in grey). The conserved amino acid motif, where cleavage of Stx2a A subunit was postulated to occur, is highlighted in yellow, whereas the located cleavage sites induced by furin (green), trypsin (blue) or components present in stool from healthy human donors (blue and red) are indicated with arrows, respectively. * Furthermore, the cleavage site potentially induced by chymotrypsin-like elastase 3B (CELA3B) is also depicted (red).

To determine the exact cleavage site of Stx2a A subunit, on the amino acid level, induced by specific enzymes or components present in human stool, Stx2a was cleaved in vitro by incubation with furin, trypsin, or stool supernatants (*n* = 7). Subsequent to Stx2a A subunit cleavage, the A1 fragment was isolated by SDS-PAGE, digested as described in Section 2.7, and sequenced by mass spectrometry. The two enzymes tested, furin and trypsin, as well as human stool component(s) induced cleavage of the Stx2a A subunit in the same amino acid region. The results confirmed that Stx2a cleavage by furin is located after the second arginine (Arg/R) of the amino acid motif R-X-X-R [19], while the cleavage caused by trypsin was located after the first Arg/R of the above-mentioned amino acid motif (Figure 4). Components present in human stool cleaved Stx2a A subunit at two distinct positions, suggesting the involvement of more than one protease. One of the positions mirrored the cleavage position of trypsin. The second position, however, appeared after the alanine (Ala/A) and before the first Arg/R of the respective motif (Figure 4). Based on this cleavage site and proteins detected in fraction 5, the serine protease CELA3B was confirmed as another potential candidate responsible for the enzymatic cleavage of Stx2a A subunit. Five out of seven investigated cleavage sites induced by SF-SN HS are compatible with a cleavage by both trypsin and CELA3B, since cleavage was found to occur after both the first Arg/R within and the Ala/A before the relevant motif R-X-X-R, respectively. The remaining two samples showed cleavage sites compatible with either trypsin or CELA3B cleavage.

### 3.4. Chymotrypsin-like Elastase 3B (CELA3B) Induces Partial Cleavage of A Subunit of Stx2a In Vitro

**Figure 5:**
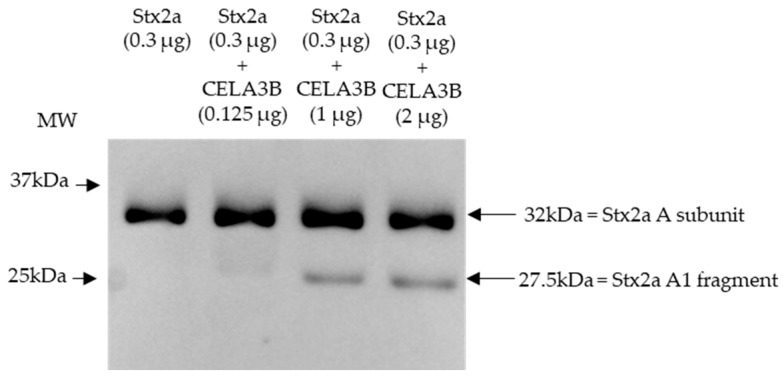
Shiga toxin 2a (Stx2a) A subunit and/or its A1 fragment after incubation with recombinant CELA3B. This representative immunoblot shows the molecular size of the A subunit or its fragments of Shiga toxin 2a (Stx2a) after incubation (2 h) of Stx2a with different concentrations of chymotrypsin-like elastase 3B (CELA3B). Pure Stx2a incubated under the same conditions without CELA3B was used as a reference. Stx2a was detected with a polyclonal mouse anti-Stx2a. Arrows indicate the Stx2a bands corresponding to the whole A subunit or the A1 fragment. Molecular weight (MW) is indicated in kilodaltons (kDa) based on the migration of the molecular marker.

Based on the cleavage position induced by components present in stool and the proteins identified in stool fractions, which induce Stx2a A subunit cleavage, in vitro experiments were performed to assess the ability of human CELA3B to cleave the Stx2a A subunit. Recombinant CELA3B was incubated with Stx2a in different concentrations and the molecular mass of the latter was subsequently analyzed by immunoblotting. Independent of the incubation time (2 or 16 h), the addition of adequate amounts of recombinant CELA3B resulted in partial Stx2a A subunit cleavage, which was detected by the identification of an additional band having a molecular mass corresponding to Stx2a A1 fragment (~28 kDa) by immunoblotting (Figure 5).

### 3.5. Stool of Healthy Donors Appear to Contain CELA3B

**Figure 6:**
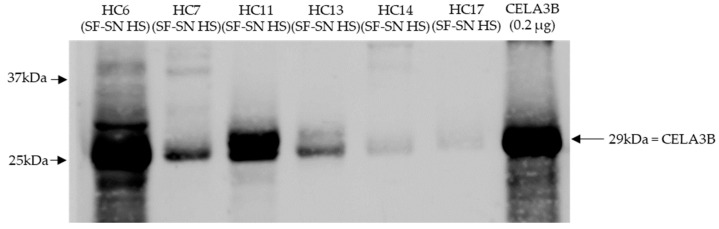
CELA3B was present in sterile-filtered supernatant of human stool derived from heathy individuals. This representative immunoblot shows the presence of chymotrypsin-like elastase 3B (CELA3B) in tested samples (*n* = 6); however, for some, this was in very low amounts (*n* = 2). Recombinant CELA3B served as reference. The band showcasing CELA3B is indicated by arrow. Molecular weight (MW) is indicated in kilodaltons (kDa) based on the migration of the molecular marker.

Stored sterile-filtered supernatant of human stool was found to contain CELA3B by Western blotting. CELA3B was detected in the majority of the tested samples (*n* = 9), possibly at different amounts (Figure 6).

### 3.6. Stool from EHEC-Infected Patients Show a Similar Stx2a Cleavage Pattern

**Figure 7:**
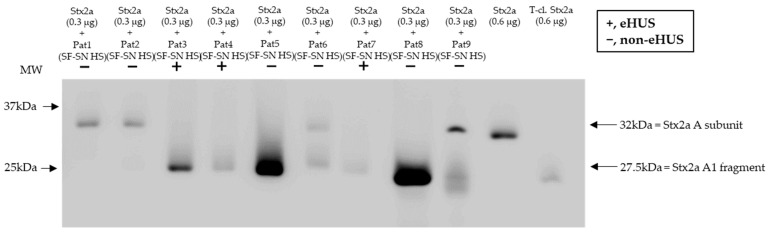
Cleavage of Shiga toxin 2a (Stx2a) by stool supernatant from patients with an EHEC infection. Immunoblots show the form (intact or cleaved) of Stx2a after its incubation (30 min) with sterile-filtered supernatant derived from human stool (SF-SN HS) of EHEC-patients (*n* = 9), including EHEC-associated hemolytic uremic syndrome (eHUS) cases (*n* = 3). Pure Stx2a and trypsin-cleaved (T-cl.) Stx2a served as references. Bands showcasing the whole A subunit or the A1 fragment of Stx2a are indicated by arrows. Molecular weight (MW) is indicated in kilodaltons (kDa) based the on migration of the molecular marker.

To confirm that Stx2a A subunit cleavage can also occur during a native infection, Stx2a was incubated with sterile-filtered supernatant of stool derived from patients suffering from an EHEC infection (*n* = 9) and experiencing varying disease severity, including bloody diarrhea and eHUS (*n* = 3). Subsequent characterization of Stx2a A subunit structure (intact or cleaved) by immunoblotting revealed that cleavage was also induced when using patient stool samples. Nonetheless, the presence or absence of cleavage did not correlate with disease severity (Figure 7). 

## 4. Discussion

It has been proven that the cleavage of the Stx A subunit is essential for most Stx subtypes to acquire the most potent toxicity [19,21,28,29]. Despite the first report presenting trypsin as the enzyme responsible for in vitro cleavage of Stx A subunit [18], it has been presumed that in vivo cleavage actually occurs intracellularly. Hereby, furin, among other serine proteases, was found to be the most prominent mediator of Stx A subunit cleavage within cells [19]. Our recent finding demonstrated that the Stx2a A subunit can also be cleaved during its purification [24], and prompted us to investigate whether Stx2a A subunit cleavage can also occur extracellularly during a natural EHEC infection, before the target cells are reached. This research question is especially important because variations in Stx2a A subunit structure impact the toxin’s biological properties. These properties include binding to human neutrophils, the induction of platelet–leukocyte complex formation, and binding to the complement regulatory protein factor H that delays the onset of its complement inhibitory effects [24]. These findings highlighted the importance of the characterization of the Stx2a structure (intact or cleaved) in related studies as well as the need for a standardized purification protocol. 

In order to analyze whether bacteria from human microbiota could induce cleavage of Stx2a A subunit, we studied the structure of the Stx2a A subunit following incubation with culture supernatants of certain bacteria found in the human microbiota, such as *Bacteroides thetaiotaomicron* or *Enterococcus faecalis*. The results, however, did not demonstrate an ability of these bacterial cultures to facilitate Stx2a A subunit cleavage. Isolated cultures in the absence of host factors may not accurately model in vivo environments, in which the interplay between the gut microbiota and host gene expression subsequently affects host pathways [30]. Thus, future research involving the human microbiome should be performed considering these complex interactions.

In the main body of this study, the structure (intact or cleaved) of the Stx2a A subunit was characterized after exposure to human stool supernatants, a noninvasive method that is thought to be quite representative of the intestinal environment. The results demonstrated that stool components from healthy individuals of different ages, including children and adults (*n* = 12), had the ability to completely cleave the Stx2a A subunit. In the same system, the addition of serine protease inhibitors, including AEBSF, Leupeptin, and Aprotinin, proved to inhibit Stx2a A subunit cleavage, while other non-serine protease inhibitors against different groups of proteases did not. The cleavage-inducing component(s) in stool was(were) proposed to belong to the family of serine proteases. Thereafter, size exclusion chromatography was performed with supernatant of healthy human stool and fraction 5, the fraction identified to be capable of cleaving the Stx2a A subunit, was then investigated using mass spectrometry. Out of a pool of hundreds of proteins identified within this fraction, trypsin was one of the most abundant. Since trypsin has previously been shown to induce Stx2 cleavage [18], it was concluded that stool-derived trypsin was the most likely candidate responsible for the observed enzymatic cleavage of Stx2a A subunit. Furin, mainly an intracellular protease that has also been found extracellularly [31], was not present in fraction 5. This supports the conclusion that furin is not the Stx cleaver present in stool.

Garred and colleagues determined that artificial cleavage by furin of Stx2a occurred after the second Arg of the amino acid motif R-X-X-R [19]. This motif was found to be located between cysteine (Cys/C) residues forming a disulfide bond which links the A subunit fragments, A1 and A2, once they are generated. In the present study, similar results were obtained by artificial induction of cleavage with trypsin. However, the cleavage was identified to occur after the first Arg/R of the R-X-X-R motif. Nevertheless, this was expected since specific cleavage locations by enzymes other than furin have already been considered [21]. Surprisingly, analysis of the exact amino acid position of the Stx2a A subunit cleavage after its incubation with human stool identified two distinct cleavage positions. Whereas one position corresponded to cleavage by trypsin (after the first Arg/R), the second occurred after Ala/A, an amino acid embedded just before the R-X-X-R motif (Figure 4). The stool fraction content identified by mass spectrometry, i.e., the list of most abundant proteins in the fraction, together with the characterized cleavage position after Ala/A, suggested the serine protease CELA3B as another potential candidate responsible for inducing Stx2a A subunit cleavage in the human intestine. O’Brien’s group previously demonstrated that mouse elastase, which is highly homologous to human CELA3B, is able to cleave the Stx2d subtype [22,23]. Their studies simultaneously showed that elastase found in human or mouse mucus enhanced the toxicity of Stx2d for Vero cells by 10- to 1000-fold [22], without affecting the cytotoxicity of Stx1, Stx2, Stx2c, and Stx2e [23]. Consequently, Melton-Celsa and co-workers hypothesized that the increased cytotoxicity of Stx2d, caused by mouse elastase, occurred due to the deletion of two amino acids at the C-terminus of the Stx2d A2 fragment by improving the binding or enhancing avidity of Stx2d to Gb3 receptors, or alternatively by influencing Stx2d intracellular trafficking [23].

The current study demonstrated that human recombinant CELA3B is capable of inducing cleavage of the Stx2a A subunit. However, with the current in vitro experimental setting, only a partial cleavage of Stx2a was achieved using recombinant CELA3B. Thus, the exact localization of the cleavage site on an amino acid level induced by CELA3B could not be confirmed and remains to be assessed in the future. It is not possible to exclude that the partially achieved cleavage of the Stx2a A subunit was related to incubation conditions, low enzymatic activity of the commercially available CELA3B (not tested by CELA3B manufacturer), or the lack of crucial cofactors, such as calcium or zinc, in the buffers [32,33]. Additionally, since relatively high concentrations of CELA3B in relation to Stx2a concentration were used, future studies need to assess whether this induction of partial cleavage also occurs under physiological conditions.

Investigations regarding the presence of the potential cleaver CELA3B in human stool supernatants led to the detection of CELA3B in all tested samples derived from healthy individuals (*n* = 9). This is not at all surprising, as human CELA3B is a pancreatic enzyme and is, as other serine proteases, involved in digestive processes within the intestine. Excretion of elastases in stool is frequently used for clinical assays in order to determine pancreatic function [34,35]. Interestingly, the amounts of CELA3B detected in individual samples seemed to differ, leading to the speculation that the amount of CELA3B present in the human intestine might potentially correlate with the extent of Stx2a cleavage. Further studies are required to prove this hypothesis.

Trypsin as well as pancreatic elastase, both present in stool and proposed to cleave Stx2a A subunit, belong to the family of serine proteases which are ubiquitously present in the host’s intestine. Therefore, cleavage of Stx2a A subunit could potentially occur directly in the gut after Stx secretion by EHEC by the cooperative action of these serine proteases. After secretion, the passage of Stx through the epithelial lining of the intestine mucosa is explained by the loosening of tight junctions caused by the intimate adhesion of EHEC and/or by the opposite transmigration of neutrophils, or by micropinocytosis-driven transcellular transcytosis [9,36,37,38]. These mechanisms seem largely independent of the binding activities of Stx2a for Gb3Cer or TLR4, suggesting that both forms of Stx2a, uncleaved and cleaved, pass the intestinal blood barrier. However, future investigations should clarify whether even cleaved Stx2a could actually pass the intestinal blood barrier.

Furthermore, stool from patients suffering from an EHEC infection (*n* = 9) showed an overall ability to cleave Stx2a A to a certain extent, independent of the patient’s clinical presentation, including bloody diarrhea and eHUS. Potential traces of blood in stool from patients neither interfered with the methodology nor influenced the results on Stx cleavage as proven by incubation of the toxin with stool of healthy donors spiked with blood. Therefore, it is proposed that the exact cleavage position, or the amount of cleavage, rather than the mere ability of a Stx2a A subunit to be cleaved, could be one of the key determinants of Stx2a pathogenicity, thereby impacting eHUS development. However, in future studies, asymptomatic patients and those with a mild disease progression should be included as control groups. Additional investigations examining potential variations between the cleaved Stx2a by various enzymes should be carried out, as cleavage at various amino acid positions may potentially affect the biological features of Stx2a, as is the case for Stx2d [22,23]. Interestingly, a report on fecal serine protease activity in inflammatory bowel disease demonstrated a 10-fold increase in trypsin-like, elastase-like, and cathepsin G-like proteolytic activity [39]. Thus, it is important to consider that the extent of Stx2a A subunit cleavage induced by a given individual could vary depending on the different concentrations of cleavage-responsible enzymes in their stool. Furthermore, the sampling conditions and time point of infections might have differed among individual patients. A novel approach that could be considered for future studies would be the use of human intestinal organoids to evaluate not only the tissue response to Stxs, as reported by Pradhan and colleagues [40], but also the Stx2a structure after contact with those organoids. Moreover, for a better understanding of the Stx2a structure in circulation, and after its interaction with host factors, the generation of monoclonal antibodies capable of discriminating between Stx2a structures (uncleaved and cleaved) would be beneficial. Since the application of antibodies against Stxs has also been discussed as therapeutic measurements [41], newly invented antibodies discriminating the structure of Stx2a might even open new potential treatment approaches. 

Since this study aimed to demonstrate that Stx2a can possibly be cleaved in the gut, pilot experiments were performed with a relatively small sample size for healthy donors (*n* = 12). However, the hypothesis was also tested on EHEC-infected patients with different disease manifestations, including bloody diarrhea and eHUS (*n* = 9). Nevertheless, to confirm that these findings actually occur during an EHEC infection, studies with larger cohorts are mandatory. These should include healthy donors and EHEC-infected patients who demonstrate an asymptomatic course of disease, in addition to diseased patients (with diarrhea (watery or bloody) or eHUS).

## 5. Conclusions

The current study presented evidence of the potential cleavage of Stx2a A subunit, complete or partial, in the gut. This extracellular event would occur much earlier than the previously described intracellular cleavage during toxin trafficking in target cells. Two serine proteases, trypsin and CELA3B, were identified as potential candidates that may trigger the extracellular cleavage of Stx2a A subunit directly after its secretion by EHEC in the host’s gut. In future, studies with larger cohorts should investigate whether this observation indeed translates to natural infections and plays a role in eHUS pathogenesis. Further research should also examine whether the host’s protease profile might determine the biological characteristics of Stx2a, which in turn could affect disease development and outcome.

## Data Availability

The electronic supplementary material provides information supporting our results. Data are available upon reasonable request to the corresponding author (R.W.).

## Supplementary Materials

The following supporting information can be downloaded at: https://www.mdpi.com/article/10.3390/microorganisms11102487/s1, Figure S1. Shiga toxin 2a (Stx2a) A subunit and/or its A1 fragment after exposure to stool specimens of healthy individual; Figure S2. Shiga toxin 2a (Stx2a) A subunit and/or its A1 fragment after exposure to stool specimens of healthy individuals in presence of serine protease inhibitors; Figure S3. Chromatogram of human stool supernatant; Figure S4. Shiga toxin (Stx) 2a A subunit and/or its A1 fragment after exposure to fractions of stool supernatant of a healthy individual; Figure S5. Molecular weight (MW) of Shiga toxin (Stx) 2a A subunit/fragment; Table S1. Top 10 proteins present in fraction 5 of the first analyzed human stool supernatant.
