# Supplementary material for: Enzymatic Cleavage of Stx2a in the Gut and Identification of Pancreatic Elastase and Trypsin as Possible Main Cleavers
Source: Microorganisms. 2023 Oct 4;11(10):2487. doi: 10.3390/microorganisms11102487 (PMC10609011; doi:10.3390/microorganisms11102487)
Supplement: Supplementary file 1 [file microorganisms-11-02487-s001.zip › microorganisms-2508491-supplementary.pdf]

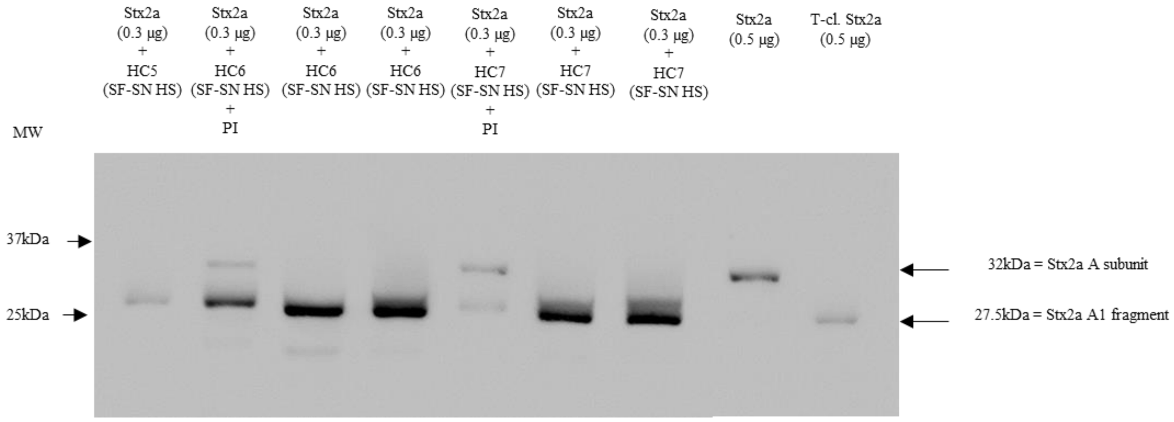

Figure S1. Shiga toxin 2a (Stx2a) A subunit and/or its A1 fragment after exposure to stool specimens of healthy individual (n=3). This immunoblot shows the form (intact or cleaved) of Stx2a after its incubation (10 min) with sterile-filtered supernatant derived from human stool (SF-SN HS) with or without a protease inhibitor cocktail (PI). Pure Stx2a and trypsin-cleaved (T-cl.) Stx2a served as references. Bands showcasing the whole A subunit or the A1 fragment of Stx2a are indicated by arrows (the A2 fragment (~4.5 kDa) would be too short to be detected). Molecular weight (MW) is indicated in kilodaltons (kDa) based on the migration of the molecular markers.

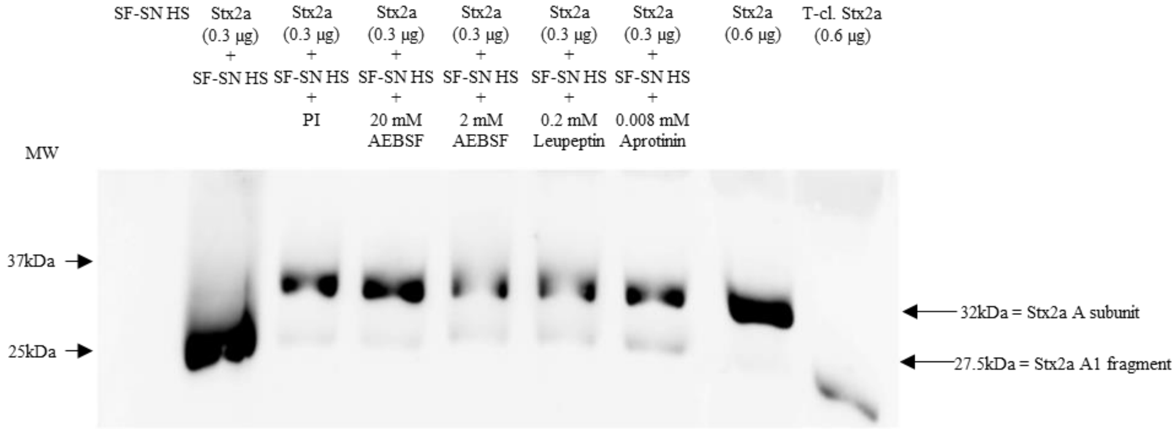

Figure S2. Shiga toxin 2a (Stx2a) A subunit and/or its A1 fragment after exposure to stool specimens of healthy individuals in presence of serine protease inhibitors. Immunoblots show the form (intact or cleaved) of Stx2a after its incubation (15 min) with sterile-filtered supernatant derived from human stool (SF-SN HS) with or without a protease inhibitor cocktail (PI), AEBSF (2 or 20 mM), Leupeptin (0.2 mM), Aprotinin (0.008 mM). Pure Stx2a and trypsin-cleaved (T-cl.) Stx2a served as references. Bands showcasing the whole A subunit or the A1 fragment of Stx2a are indicated by arrows. Molecular weight (MW) is indicated in kilodaltons (kDa) based the on migration of the molecular markers.

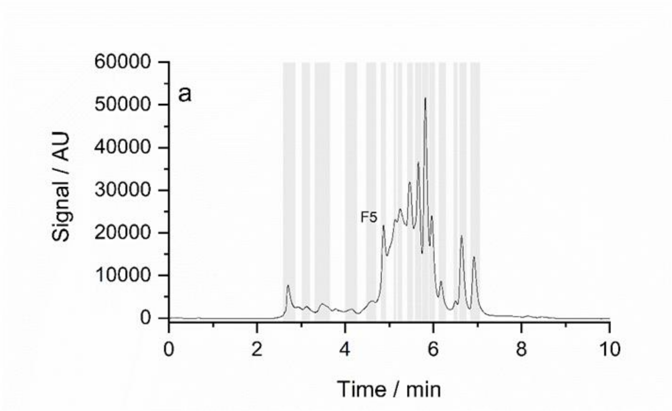

Figure S3. Chromatogram of human stool supernatant. Exemplary chromatogram of a fractionated stool supernatant sample. The collected fractions are highlighted in gray.

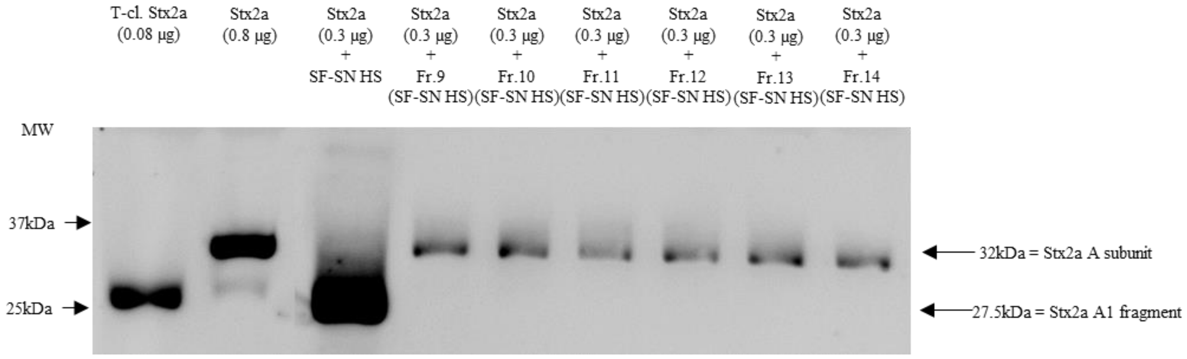

Figure S4. Shiga toxin (Stx) 2a A subunit and/or its A1 fragment after exposure to fractions of stool supernatant of a healthy individual. Immunoblots show the form (intact or cleaved) of Stx2a after its incubation (30 min) with selected fractions (3 to 8) of sterile-filtered supernatant derived from human stool (SF-SN HS). Pure Stx2a and trypsin-cleaved (T-cl.) Stx2a served as references. Bands associated with the whole A subunit or the A1 fragment of Stx2a are indicated by arrows. Molecular weight (MW) is indicated in kilodaltons (kDa) based the on migration of the molecular markers.

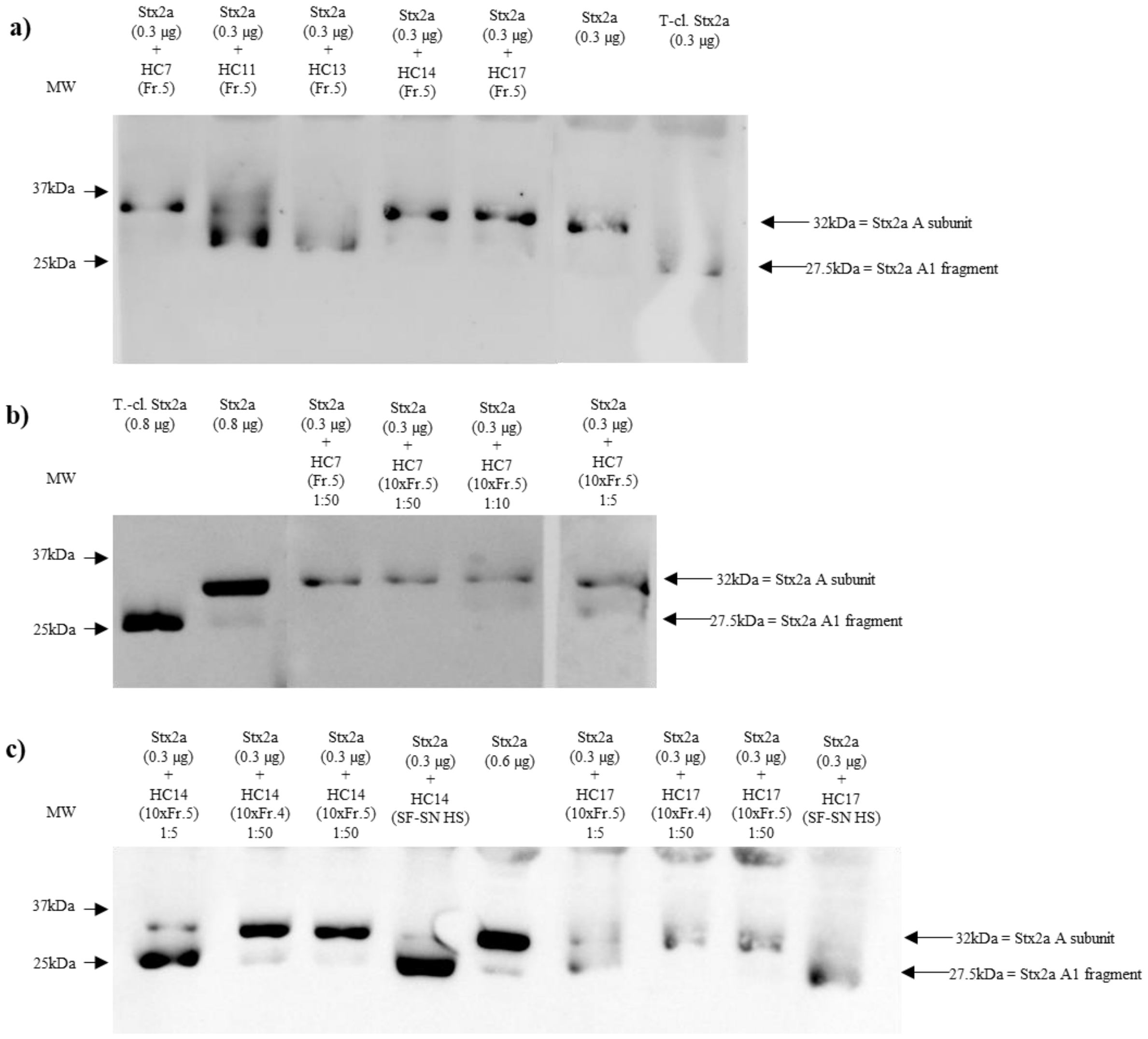

Figure S5. Molecular weight (MW) of Shiga toxin (Stx) 2a A subunit/fragment. **a)** after exposure to fraction 5 of stool supernatant of remaining tested healthy individuals (n=5), and after exposure to concentrated fraction 5 derived from **b)** healthy control (HC) 7 and **c)** HC14 and HC17. This immunoblot shows the structure of Stx2a after its incubation (30 min) with fraction (Fr.) 5 or concentrated Fr.5 (10x) of sterile-filtered supernatant derived from human stool (SF-SN HS). Pure Stx2a and trypsin-cleaved (T-cl.) Stx2a served as references. Bands showcasing the whole A subunit or the A1 fragment of Stx2a are indicated by arrows. MW is indicated in kilodaltons (kDa) based on the migration of the molecular markers.

Table S1. Top 10 proteins present in fraction 5 of the first analyzed human stool supernatant. Mass spectrometry was performed on the proteins present in fraction 5 of human stool supernatant fractionated by gel-filtration. The 10 most abundant proteins, of several identified are listed.

Abundance	Protein
1	Carboxypeptidase A1
2	Carboxypeptidase B
**3**	**Chymotrypsin-like elastase 3B (CELA3B)**
4	Lithostathine-1-alpha
5	Immunoglobulin heavy constant alpha
6	Deleted in malignant brain tumors 1 protein
7	Keratin, type II cytoskeletal 1
8	Cadherin-related family member
**9**	**Trypsin-2**
10	Keratin, type I cytoskeletal 9
